# Sleep traits and associated factors among people living with HIV/AIDS in Iran: a two-step clustering analysis

**DOI:** 10.1038/s41598-024-53140-x

**Published:** 2024-03-01

**Authors:** Safieh Mohammadnejhad, Arezu Najafi, Valerie A. Earnshaw, Mohammad Ebrahimzadeh Mousavi, Akbar Fotouhi, Samaneh Akbarpour

**Affiliations:** 1 Department of Epidemiology and Biostatistics, School of Public Health, Tehran University of Medical Sciences, Tehran, Iran; 2 Occupational Sleep Research Center, Baharloo Hospital, Tehran University of Medical Sciences, Tehran, Iran; 3 Department of Human Development and Family Sciences, University of Delaware, Newark, DE USA; 4 Department of Clinical Psychology, Faculty of Behavioral Science, University of Social Welfare and Rehabilitation Sciences, Tehran, Iran; 5 Sleep Breathing Disorders Research Center (SBDRC), Tehran University of Medical Sciences, Tehran, Iran

**Keywords:** Diseases, Health care, Risk factors

## Abstract

Sleep plays an essential role in improving the quality of life of people living with HIV (PLWH); however, sleep traits in this population are not well studied. This study aims to evaluate the sleep traits and related associated factors among PLWH in Iran. A nationwide cross-sectional study was conducted with 1185 PLWH who attended Voluntary Counseling and Testing centers in 15 provinces in Iran between April 2021 and March 2022. The Berlin Obstructive Sleep Apnea questionnaire, Pittsburgh Sleep Quality Index, Epworth Sleepiness Scale and Insomnia Severity Index were used. A two-step clustering method was employed to identify the number of sleep clusters in PLWH. Prevalence of poor sleep quality, sleepiness and insomnia were 49.6%, 21.15% and 42.7% respectively. Three sleep trait clusters were identified: I. minor sleep problems (45.6%); II. Snoring & sleep apnea (27.8%), and III. poor sleep quality and insomnia (26.7%). Age (Odds Ratio (OR) 1.033, 95% Confidence Interval (CI) 1.017–1.050), academic education (OR 0.542, 95% CI 0.294–0.998) and HIV duration were associated with being in Snoring & sleep apnea cluster, while age (OR = 1.027, 95% CI 1.009–1.040) was associated with being in Poor sleep quality and insomnia cluster. PLWH with depression had higher odds of being in Poor sleep quality and insomnia cluster, and those with anxiety had higher odds of being in Snoring & sleep apnea cluster and Poor sleep quality and insomnia cluster. A significant proportion of PLWH have poor sleep quality, sleepiness, and insomnia. The identification of three distinct sleep trait clusters underscores the need for increased attention and tailored interventions to address the specific sleep issues experienced by PLWH.

## Introduction

Sleep is one of the most important around the clock cycles in the human body system, which plays an important role in physiological and psychological functions. Disturbances to the sleep–wake cycle, such as sleep apnea^1^, insomnia^2^, poor sleep quality^3^ and sleepiness^4^, can negatively impact physical and social performance, thereby undermining the quality of life. People living with HIV (PLWH) are particularly susceptible to sleep disorders, which can adversely affect their health and quality of life. However, several studies have already shown PLWH experience sleep disorders, which substantially affect their physical and social performance^5–7^. Greater understanding of sleep problems among PLWH can lead intervention efforts to improve sleep and ultimately, quality of life of PLWH.

Iran is one of 30 fast-track countries identified by UNAIDS, accounting for 89% of all new HIV infections globally^8^. There have been more than 54,000 cases^9^ of PLWH in Iran so far. Sleep disturbances are prevalent in PLWH in Iran, with up to 75% of adults estimated to be affected^10–13^. A study conducted in Iran found that 72% of PLWH suffered from poor sleep quality. Also, in another study, 55.3% of PLWH had sleep disturbances^10^. Despite several studies on the prevalence of sleep problems in PLWH in Iran, to our knowledge, no nationwide studies have evaluated sleep traits using cluster analysis.

In recent years, researchers believe that some similar diseases or associated factors are not randomly distributed in the population and occur in combination with others that can be expected with higher risk from each of the associated factors alone. Sleep problems including various clinical items as poor sleep quality, excessive daytime sleepiness, insomnia, and obstructive sleep apnea (OSA) is one of the known conditions that tend to occur simultaneously in some specific populations. Identification of different sleep problem clusters’ (traits) and the related factors in the PLWH can help identify high-risk subgroups for targeted interventions.

Different methods are utilized in scientific sources to cluster individuals. A cluster refers to a group of people or objects that are gathered together based on similarities, while they are less similar to other clusters. In this study, the two-step clustering method was selected after assessing the compatibility of the data with existing methods. Identifying clusters of health-related problems or behaviors assists in recognizing factors that commonly co-occur, as well as factors associated with these clusters. In this research, our objective is to identify distinct patterns of sleep problems that differ from each other but are similar in each subgroup. Since we acknowledge that these similarities and differences are not by chance, we will investigate the main reasons behind their existence. Consequently, we can develop and implement the most effective intervention strategies to reduce or eliminate sleep problems within any subgroup of HIV patients. Given the multitude of diseases and sleep problems experienced by these patients, our approach allows us to bring about positive improvements in their quality of life by effectively managing a range of sleep-related challenges.

Although there are some studies on the prevalence of sleep problems in PLWH in Iran^10,13,14^, no studies to date have evaluated sleep traits among a nationwide sample of PLWH using cluster analysis. Therefore, this study aimed to identify sleep traits and related associated factors among PLWH in Iran using a two-step clustering method. The findings from this study can inform tailored interventions to improve sleep and enhance the quality of life of PLWH in Iran.

## Methods and materials

### Study population and sample size

This national cross-sectional study included a sample of 1185 adults aged 18 years or older with confirmed HIV/AIDS more than three months that was conducted between April 2021 and March 2022. To allocate the appropriate sample size to each province, a random selection process was utilized. Three provinces were randomly selected from across each geographical region of Iran (North-East, North-West, Central, South-East, and South-West), resulting in a total of 15 province. The total sample size was then distributed proportionally based on the number of diagnosed patients in each province. Afterward, a compilation of Voluntary Counseling and Testing Centers (VCTs) was made for each of the 15 provinces. Two VCTs were subsequently randomly chosen from these lists in each province, serving as the source for data collection. Finally, convenience sampling was employed in each VCT to select participants.

Of the initial sample of 1212 PLWH, 27 participants had missing data on sleep variables and were excluded from the analysis.

Skilled interviewers with experience working with PLWH were completed the study questionnaires. We ensured them by explaining the purpose of the study and guaranteeing confidentiality of the information, and then receiving their informed verbal consent. This study has a code of ethics from the research ethics committee, School of Public Health, Tehran University of Medical Sciences, Tehran, Iran under the number IR.TUMS.SPH.REC.1400.009.

### Tools, measurements, and data collection

Data collection was included two components of physical examination and completion of several questionnaires. Several validated questionnaires were used to assess sleep traits, including the Pittsburgh Sleep Quality Index (PSQI)^16^, the Berlin Obstructive Sleep Apnea Questionnaire (OSAQ)^15^, the Epworth Sleepiness Scale (ESS)^17^, the Insomnia Severity Index (ISI)^18^. In addition, demographic information (such as age, gender, education, marital status, occupation) and the disease-related information (such as disease duration, high risk behaviors at the time of diagnosis, hepatitis C virus, hepatitis B virus, and tuberculosis (TB)) were extracted through questions from patients or the participants' clinical records. Viral load and CD4 count data were extracted from the participants' clinical records. The clinical examinations measuring height and weight were performed during the interview session. Weight was measured with little clothing and without shoes by Using the digital scale available in the centers. Height was measured without shoes by using a meter. Body Mass Index (BMI) was calculated by dividing weight (kg) with the square of height (m)^15^.

- *PSQI* The PSQI is a 19-item questionnaire with seven subscales that measures subjective sleep quality, sleep latency, sleep duration, sleep efficiency, sleep disturbances, use of sleeping medication, and daytime dysfunction. The PSQI also measures time going to bed, fall asleep, waking up, and duration of sleep. A score above 5 indicates poor sleep quality, and the Persian version of the questionnaire has a reliability of 0.89 and a validity of 0.86^16^.
- *OSAQ* The OSAQ is a 10-item questionnaire with three subscales that assess the risk of developing sleep apnea. A positive score in more than one subscale out of the three indicates a high risk of developing sleep apnea^17^. The validity and reliability of this tool have been confirmed with Cronbach's alpha coefficient of 0.92^18^ for the original version and 0.90 for the Persian version^19^.
- *ESS* The ESS is an 8-item questionnaire that measures sleepiness levels in everyday situations and has a total score ranging from 0 to 24. A score of 10 or higher indicates severe daytime sleepiness^20^. In the study of Sadeghniiat Haghighi and et al. the questionnaire's reliability was confirmed with a Cronbach's alpha of 0.81. Additionally, the validity of the questionnaire was noted to be favorable in terms of its content^21^.
- *ISI* The ISI is a seven-item questionnaire that assesses the difficulty of falling asleep, staying asleep, waking up and the person's satisfaction with the structure of recent sleep, and the impact of sleep problems on personal life and daily functioning. The overall score ranges from 0 to 28, with scores of 8–14 indicating a mild disorder, 15–21 indicating moderate clinical insomnia, and 22–28 indicating severe clinical insomnia^22^. In the Persian version of the ISI questionnaire, the internal consistency demonstrated by Cronbach's alpha coefficient was above 0.8 and the intra-class correlation coefficient was above 0.7^23^.
- *Depression, anxiety, and stress questionnaire (DASS)* The DASS was used to measure the severity of depression, anxiety, and stress symptoms. The Persian version of the questionnaire has been validated and has a high level of reliability, with Cronbach's alpha coefficients of 0.81, 0.74, and 0.78 for the depression, anxiety, and stress scales, respectively^24^. The questionnaire consists of 21 items, with each scale comprising seven questions. Each question is scored from 0 to 3. Questions 21, 17, 16, 13, 10, 5, and 3 are for depression, and a score above 9 indicates that the person has one of the depressive states. Questions 20, 19, 15, 9, 7, 4, and 2 are for anxiety, and a score above 7 indicates having one of the anxiety states. Also, questions 18, 14, 12, 11, 8, 6, and 1 are for stress disease, and a score above 14 is a sign of having one of the stress states^25^.

### Statistical analysis

The initial sample consisted of 1212 PLWH of which 27 participants had missing data on sleep variables and were excluded from the analysis. Approximately 3.01% of the cells in the dataset (from 1,185 participants) had missing data, which were imputed using single imputation and regression models. All subsequent analyses were performed using survey methods to address cluster sampling and sampling weight. The data were weighted according to the 2021 national Iranian PLWH aged ≥ 15 years, categorized by age and sex. Weighted means with Standard Error (SE) and weighted percentages were used to report the continuous and categorical variables. In the survey method, we utilized ANOVA analysis (α = 0.05) to investigate differences in the quantitative baseline information and sleep traits across participant clusters, and a chi-square test was employed for qualitative variables.

A two-step clustering method was employed to identify the number of sleep clusters in PLWH, considering quantitative variables (including bedtime, time to fall asleep, waking up time, sleep duration and the total scores of PSQI, ISI, and ESS) and qualitative variables (including OSAQ, snoring and puffing, difficulty falling asleep, staying asleep, and waking up in the morning) as main sleep characteristics. Initially, an automatic method was used to determine the number of clusters, resulting in five clusters. However, after examining the clusters, it was found that two of them were identical to other clusters, so the number of clusters was manually adjusted to three. The final analysis included seven continuous and five categorical variables, and the silhouette index, which was 0.2 in this study, indicated the appropriateness of the number of clusters.

Additionally, multinomial logistic regression was employed to investigate the relationship between baseline variables and sleep clusters. For the univariable analysis, the results show that the variables of age, sex, education, drug injection transmission, sexual behaviors, Disease duration, CD4 count at the time of diagnosis, Coinfections noncommunicable, as well as psychological diseases of depression and anxiety have a statistically significant relationship with the clusters with *p*-value < 0.2 and were entered in multiple models as final model. For the univariable analysis, variables with *p*-values < 0.2 were entered into multiple models as the final model.

All analyses were performed using STATA version 17 (stataCorpLLC 4905 Lakeway Drive College Station, TX 77845 USA), for complex survey analysis and *p*-value of less than 0.05 was considered statistically significant.

### Ethical approval

Informed consent is an essential part of clinical research, and it should be obtained in a way that ensures the confidentiality, comprehension and voluntariness of study participants. Since the participants in this study were PLWH, they were worried about disclosure of their sensitive information if they were obligated to sign a written consent form. Therefore, in order to solve their concern, we ensured them by explaining the purpose of the study and guaranteeing confidentiality of the information, and then receiving their informed verbal consent. Also, it is important to note that ethical considerations may vary depending on the specific context and guidelines of the research institutions. In Iran, a legal guardian is required for children under 18 years of age. However, in our study, all participants were over 18 years of age, so the requirement for a legal guardian was not applicable. Regarding the confidentiality of HIV disease, it is important to respect the privacy and confidentiality rights of patients. If some patients have not given permission to disclose their condition to their families, obtaining informed consent from parents or legal guardians could violate their rights. All interview procedures were carried out according to the relevant regulations. Our study did not include human trials. The study was approved by the ethics committee of Tehran University of Medical Sciences under the code IR.TUMS.SPH.REC.1400.009.

## Results

The analysis included a total of 1185 PLWH, with a mean age of 35.36 (SE:0.062) years. Of these, 80.66% were men including 10 transgender PLWH who were classified as men. Additionally, 42.8% of the participants were single, and 49.96% were housewives or students or unemployed without a job or income. Baseline information on the participants is presented in Table 1.

**Table 1:** Baseline information of participants (n:1185).

Variables	Weighted mean*	Standard error
Age	35.36	0.062
BMI	23.35	0.18

Variables	Frequency	Weighted proportion*
Sex		
Male	717	80.66
Female	468	19.34
Education		
Illiterate to middle school	754	50.21
High school	342	36.72
College	89	12.07
Marital status		
Single	264	42.80
Married	614	41.59
Divorced or widowed	307	15.61
Occupation		
Employee	39	2.71
Free lancer	338	35.96
Worker	113	11.38
Other (student/housewife/no job)	695	49.96

*The weighted average and proportions are based on the population of the latest HIV/AIDS reports in the first six months of 2020.

As shown in Table 2, the average duration of HIV/AIDS infection among the participants was 10.12 years. The most common high-risk behavior was drug injection (33.51%), followed by sexual behavior (heterosexual; 31.44%). Mother-to-child transmission and occupational exposure had the lowest percentage among high-risk behaviors (0.55% and 1.18% respectively). The most common infectious disease simultaneously with HIV/AIDS was hepatitis C infection, with a percentage of 13.94%.

**Table 2:** Disease characteristics among PLWH in the study.

Variables	Weighted mean^§^	Standard error
Disease duration, year	10.12	25.33

Variables	Frequency	Weighted proportion*
CD4 count at the time of diagnosis		
CD4 < 500	343	31.75
CD4 ≥ 500	842	68.25
Initial viral load		
Undetectable	494	38.72
Detectable	691	61.28
High risk behaviors at the time of diagnosis		
Drug injection	404	33.51
Sexual behavior (homosexual)	87	19.24
Sexual behavior (heterosexual)	314	31.44
Blood injection	15	1.09
Mother to child	9	0.55
Spouse of a high-risk person	68	3.07
Spouse of an affected person	330	14.79
Job exposure	14	1.18
Unknown	159	12.66
Co-infection with tuberculosis	84	5.33
Co-infection with hepatitis B	34	2.35
Co-infection with hepatitis C	193	13.94
Co-infection with tuberculosis, hepatitis B and hepatitis C diseases	264	18.25

^§^The weighted average and proportions are based on the population of the latest HIV/AIDS reports in the first six months of 2020.

Table 3 shows that 49.54% (n:587) of the participants had poor sleep quality, while 45.06% (n:503) and 25.49% (n:302) experienced insomnia and sleepiness, respectively. Moreover, 10.17% (n:154) of the PLWH suffered from OSA.

**Table 3:** Sleep traits of study participants in each cluster.

Variables	Total	First cluster (n:540)	Second cluster (n:329)	Third cluster (n:316)	*p*-value*
Time of going to bed^a^, mean ± SE	23.23 ± 0.17	23.88 ± 0.090	20.64 ± 0.54	24.65 ± 0.17	< 0.001
Time to fall asleep^b^, mean ± SE	24.48 ± 1.29	18.60 ± 1.06	19.07 ± 1.31	41.58 ± 3.55	< 0.001
Time of waking up^a^, mean ± SE	8.9 ± 0.16	7.98 ± 0.11	5.40 ± 0.48	8.11 ± 0.21	< 0.001
Sleeping duration^a^, mean ± SE	7.33 ± 0.07	7.53 ± 0.09	7.73 ± 0.12	6.53 ± 0.17	< 0.001
PSQI total score, mean ± SE	5.26 ± 0.19	3.53 ± 0.17	4.68 ± 0.21	9.20 ± 0.39	< 0.001
Insomnia index total score, mean ± SE	7.59 ± 0.31	4.68 ± 0.30	6.28 ± 0.40	14.60 ± 0.55	< 0.001
Berlin total score, mean ± SE	6.69 ± 0.25	5.39 ± 0.33	8.13 ± 0.50	7.71 ± 0.46	< 0.001
					< 0.001
Snoring, n (%)					
Yes	380 (28.04)	0	271 (75.53)	109 (33.26)	< 0.001
No	805(71.96)	540 (100)	58 (24.47)	207 (66.74)	
Trouble falling sleep, n (%)					
No	482 (42.44)	299 (57.21)	161 (48.67)	22 (7.58)	< 0.001
Mild	252 (21.22)	146 (26.28)	95 (29.9)	11 (2.46)	
Moderate	260 (19.26)	72 (8.9)	60 (15.85)	128 (42.72)	
Severe	125(10.68)	17 (4.37)	10 (3.14)	98 (30.67)	
Very sever	66 (6.39)	6 (3.24)	3 (2.44)	57 (16.56)	
Difficulty staying asleep and waking up frequently, n (%)					
No	450 (40.65)	309 (58.65)	130 (41.75)	11 (4.92)	< 0.001
Mild (2 times)	388 (32.68)	209 (37.38)	148 (44.77)	31 (11.05)	
Medium (2–3 times)	218 (16.24)	18 (3.65)	41 (10.29)	159 (46.65)	
Severe (3–5 times)	83 (5.12)	4 (0.33)	7 (0.87)	72 (18.76)	
Very severe (> 5 times)	46 (5.31)	0	3 (2.32)	43 (18.62)	
Difficulty in waking up, n (%)					
No	457 (44.85)	274 (56.79)	146 (48.98)	37 (17.6)	< 0.001
Mild	317 (25.56)	155 (25.25)	101 (29.83)	61 (21.71)	
Medium	270 (16.91)	83 (11.59)	62 (13.73)	125 (30.44)	
Severe	108 (10.12)	21 (5.77)	14 (4.42)	73 (24.4)	
Very severe	33 (2.56)	7 (0.6)	6 (3.03)	20 (5.85)	
Berlin overall score, n (%)					
High risk for apnea	154 (10.17)	0 (0)	97 (25.92)	57 (13.32)	< 0.001
Low risk for apnea	1039 (89.93)	540 (100)	232 (74.08)	259 (86.68)	
PSQI (Poor sleep)					
Yes	587(49.54)	153(28.33)	150(45.59)	284(89.87)	< 0.001
No	598(50.46)	387(71.67)	179(54.41)	32(10.13)	
ESS (Sleepiness)					
Yes	302(25.49)	90(16.67)	102(31)	110(34.81)	< 0.001
No	883(74.51)	450(83.33)	227(69)	206(65.19)	

^a^Measured in hours.

^b^Measured in minutes.

Average silhoeutte: 0.2

*ANOVA analysis for the quantitative and chi-square test for the qualitative variables.

PLWH were classified into three clusters using the two-step clustering method. The characteristics of PLWH in each cluster are reported in Table 3 which are as follows:

I. Cluster 1: Minor sleep problems—PLWH with good sleep quality, who do not wake up frequently during sleep and do not experience sleepiness during the day. The average sleep duration for this cluster was 7.53 h, and they do not suffer from snoring or sleep apnea. This cluster comprises approximately 45.6% (n:540) of the study sample.
II. Cluster 2: Snoring & sleep apnea—PLWH with some degree of sleep apnea and snoring at night. This cluster of PLWH wakes up very early in the morning, has mild degrees of poor sleep quality, and moderate degrees of sleepiness. The average sleep duration for this cluster was 7.73 h, and they make up 27.8% (n:329) of the total sample.
III. Cluster 3: Poor sleep quality & insomnia—PLWH with poor sleep quality, who sleep later at night, take longer to fall asleep, and wake up in the morning with difficulty. The average sleep duration for this cluster was 6.53 h, and they may wake up several times during the night. They also experience moderate insomnia. This cluster constitutes 26.7% (n:316) of the sample.

Table 4 shows that age, sex, education and possible transmission route differed significantly among the three clusters. Cluster 1 had the youngest average age (33.98 years), while Clusters 2 and 3 were almost similar, with an average age of 37.31 and 36.01 years, respectively. In all three clusters, there were more men than women, and most of the PLWH had no education or only middle school education. However, academic (college) education was more prevalent in Cluster 1 (17.24%), while high school secondary education and diploma were more common in Cluster 3 (44.56%). The CD4 count > 500 prevalent in Cluster 3 (76.39%), while the CD count < 500 more common in Cluster 2 (36.9%) and Cluster 1 (33.17%). No significant differences were found between the three clusters regarding occupation and marital status.

**Table 4:** The baseline information of participants in each cluster.

Variables	First cluster (n:540)	Second cluster (n:329)	Third cluster(n:316)	p-value^¥^
Age, mean ± SE	33.98 ± 0.41	37.31 ± 0.57	36.01 ± 0.80	< 0.001
BMI, mean ± SE	23.43 ± 0.27	23.40 ± 0.34	23.15 ± 0.33	0.09
Sex				
Male	312 (78.59)	218 (84.72)	187 (82.43)	0.03
Female	228 (22.41)	111 (15.28)	129 (17.57)	
Education				
Illiterate to middle	326 (44.23)	224 (61.97)	204 (49.58)	< 0.001
High school	164 (38.51)	88 (29.68)	90 (44.56)	
College	50 (17.24)	17 (8.34)	22 (5.85)	
Occupation				
Clerk	26 (3.91)	8 (1.83)	5 (1.27)	0.21
Free lancer	53 (11.18)	32 (14.38)	28 (8.61)	
Worker	146 (34.38)	115 (40.77)	77 (34)	
Other	315 (50.51)	174 (43)	206 (56.1)	
Marital status				
Single	121 (41.73)	66 (40.75)	77 (47)	0.77
Married	288 (42.7)	173 (42.09)	153 (38.91)	
Divorced or widowed	131 (15.55)	90 (17.15)	86 (14.08)	
CD4 count at the time diagnosis				
CD4 < 500	170 (33.17)	99 (36.9)	74 (23.61)	0.064
CD4 ≥ 500	370 (66.83)	230 (63.1)	242 (76.39)	
Initial viral load				
Undetectable	236 (42.92)	132 (37.06)	126 (32.3)	0.13
Detectable	304 (57.08)	197 (62.94)	190 (67.7)	
Disease duration, mean ± SE	10.45 ± 31.51	9.15 ± 39	10.48 ± 86.09	0.13
High risk behavior				
Drug injection	163 (26.51)	119 (41.65)	122 (38.67)	0.003
Sexual behaviors	290 (65.63)	168 (56.28)	147 (68.03)	0.09
Unknown	72 (14.82)	36 (12.57)	37 (8.5)	0.14
Blood injection, mother to child, job exposure	15 (4.4)	6 (1.05)	10 (1.42)	0.2
Co-infection with tuberculosis	33 (4.21)	22 (6.48)	29 (6.3)	0.3
Co-infection with hepatitis B	11 (2.22)	8 (1.43)	15 (3.35)	0.37
Co-infection with hepatitis C	71 (11.65)	63 (17.1)	59 (15.1)	0.18
Co-infection with tuberculosis, hepatitis B and hepatitis C diseases	102 (15.4)	77 (21.06)	85 (20.86)	0.14

*Fisher exact test.

^¥^ANOVA analysis for the quantitative and chi-square test for the qualitative variables.

Table 5 presents the results of the multiple multinominal logistic regression analysis examining the relationship between PLWH’s characteristics and individuals in each sleep cluster. Age was associated with individuals in Cluster 2 (OR 1.033, 95% CI 1.017–1.050) and Cluster 3 (OR 1.027, 95% CI 1.009–1.040), and participants with college education were less likely to be in Cluster 2 (OR 0.542, 95% CI 0.294–0.998) compared to Cluster 1. Participants with longer disease duration were more likely to be in Cluster 1 than Cluster 2 (OR 0.984, 95% CI 0.991–0.999). Also, PLWH with depression had 2.059 times higher odds of being in Cluster 3, while PLWH with anxiety had 1.958- and 5.674-times higher odds of being in Cluster 2 and Cluster 3, respectively (Table 5).

**Table 5:** Association between sociodemographic or disease characteristics and sleep clusters using a multinomial logistic model.

Variables (reference level)	Multivariable analysis RRR (95% CI)			
	Second cluster	*P*-value	Third cluster	*P*-value
Age	1.033 (1.017–1.050)	< 0.001	1.027 (1.009–1.040)	0.003
Sex (female)	1.493 (1.038–2.147)	0.030	0.811 (0.541–1.217)	0.312
Education (illiterate)				
High school	0.916 (0.659–1.272)	0.601	1.120 (0.784–1.601)	0.532
College	0.542 (0.294–0.998)	0.049	0.940 (0.507–1.745)	0.907
Drug injection	1.197 (0.782–1.833)	0.406	0.898 (0.556–1.451)	0.663
Sexual transmission	0.992(0.707–1.394)	0.967	1.201(0.829–1.741)	0.331
Disease duration, year	0.984(0.991–0.999)	0.03	0.992 (0.961–1.024)	0.642
CD4 count	0.911 (0.667–1.246)	0.563	1.385 (0.971–1.974)	0.072
Coinfection noncommunicable	0.979(0.663–1.446)	0.919	0.830(0.545–1.264)	0.387
Depression (normal)	1.163 (0.809–1.671)	0.413	2.059 (1.345–3.151)	0.001
Anxiety (normal)	1.958 (1.354–2.830)	< 0.001	5.674 (3.803–8.467)	< 0.001

Cluster number 1 is defined as reference.

## Discussion

This study aimed to investigate the sleep traits of PLWH at a national level in Iran. The results revealed a high prevalence of poor sleep quality, insomnia, sleepiness, and obstructive sleep apnea among PLWH. Based on the participants' sleep characteristics, they were divided into three clusters. Age, gender, education and duration of infection showed a significant relationship with the placement of PLWH in these clusters.

The prevalence of poor sleep quality among PLWH in this study was 49.54%, which is consistent with studies in Iran^26^ and other countries^27,28^. However, studies in Ethiopia, Mexico, and the USA have reported higher rates^29–31^. Among the factors affecting poor sleep quality in these PLWH, we can mention the inherent characteristics of HIV infection, stress and stressful life events^32^, and fear caused by stigma^30^.

Regarding insomnia, 45.06% of participants reported insomnia problems, which is consistent with studies in the USA with 48% and France with 50%^33,34^. The onset of insomnia is often associated with acute events, such as illness, stressful life events, or the use of sleep-disrupting medications, frequently observed in people with HIV. Problems caused by insomnia can increase stress related to the disease, compliance to treatment, and lead to more fear and anxiety among PLWH^35,36^.

The results of the ESS in this study showed that 25.49% of PLWH in Iran suffer from sleepiness. Limited research exists in Iran to investigate this issue, but studies in USA^37^, England^36^, and France^38^ have reported higher prevalence rates 29.5%, 46% and 40% respectively. Sleepiness can be a side effect of drugs taken by PLWH, fatigue, or other sleep disorders such as OSA, insomnia, poor sleep quality, or psychiatric conditions like depression.

This study found that 10.17% of PLWH are at high risk of OSA, which is inconsistent with another study in Iran, with a prevalence of 48.7%^14^. However, some other studies have reported higher prevalence rates of sleep apnea among PLWH in different parts of the world^39,40^. The high prevalence of this problem may be caused by antiviral drugs, reduced physical activity, and inflammation caused by the disease^41–43^. The most crucial difference between obstructive sleep apnea and other sleep-related disorders is the obstruction of the respiratory airways, which can play a vital role in this disease, which increases the importance of screening for this problem in the PLWH.

In this study, PLWH were classified into three clusters based on sleep-related factors, which showed a significant relationship with age, gender, education and duration of infection. Although no cluster study has been conducted on PLWH to compare the results, various studies have shown a relationship between these variables and sleep disorders^44,45^. The first cluster was the largest, comprising 45.6% of the sample and including participants with fewer sleep problems than the other two clusters. Examining the characteristics of this cluster suggests that the time of going to bed, waking up, and the total duration of sleep is within the normal range of healthy individuals.

The second cluster, comprising approximately 27.8% of the study population, was found to have a high risk for obstructive sleep apnea, with snoring and sleepiness tending to cluster in these individuals. While 75% of people in this cluster reported snoring, about 30% experienced daytime sleepiness. People in this cluster also tended to go to bed earlier at night and wake up very early in the morning, which may prevent them from experiencing deep sleep due to disturbed REM sleep^46^. Age has also been found to be a factor related to obstructive sleep apnea, and the shorter duration of the disease was positively associated with the placement of individuals in this cluster. This could be related to the use of drugs to reduce the side effects of the disease, followed by the reduction of inflammation caused by the disease^47,48^.

The third cluster, comprising approximately 26.7% of the study population, was found to experience longer sleep onset latency due to insomnia. Only 7% of people in this cluster reported no difficulty in falling asleep, indicating that the majority of individuals in this cluster struggle with initiating sleep. Multinomial logistic regression analysis revealed that age, depression, and anxiety were significantly associated with the placement of individuals in this cluster. Various studies have shown that with increasing age, insomnia tends to increase, and the quality of sleep becomes poorer in people^49^, including PLWH^50^.

This study provides valuable insights into the clustering of sleep disorders and associations with mental health issues among PLWH in Iran. However, the contextual factors unique to Iran, including difference in sociocultural attitudes, healthcare access, and stigma, may limit the generalizability of these findings to other populations. Further research is warranted to explore the generalizability of these findings to diverse cultural and regional contexts, emphasizing the need for caution when applying the study’s conclusions beyond the specific context of HIV/AIDS in Iran.

Based on the information provided, policymakers should give greater attention to the following aspects. First, it’s the better for they to focus on increasing awareness and implementing standardized screening protocols for sleep disorders. They should also put effort into establishing an appropriate referral system during clinical examinations. Furthermore, policymakers must prioritize funding for research on the specific needs and challenges related to sleep disorders and mental health in PLWH. This financial support can be instrumental in supporting the development of interventions and treatments that are Appropriate to this particular population.

To date, no study has investigated the sleep and mental health disorders by clustering analysis in Iran’s population. Therefore, it is advisable to undertake a national study to compare the findings in two groups: PLWH and People without HIV. Additionally, the study took place during the COVID-19 pandemic, it would be beneficial to replicate this research following the conclusion of the pandemic and compare the results from both studies.

## Limitations and strengths

Data collection for the current study was conducted during the COVID-19 pandemic, with many PLWH hesitant to visit the VCT due to fears of contracting the virus. Therefore, random sampling was not feasible, and convenience sampling was employed.

While the pandemic may have influenced the prevalence of sleep disorders among PLWH, it could also be considered a strength of the study, as it sheds light on the experiences of PLWH during this challenging time. However, it is essential to replicate the study in non-pandemic periods to determine the generalizability of the findings.

This study is the first national research on sleep-related factors of PLWH in Iran, a UNAIDS fast-track country. The data collection was carried out by trained and skilled health workers in behavioral counseling centers and monitored by experts, ensuring the quality of the data.

## Conclusion

In conclusion, the results of the present study indicate that OSA tends to cluster with snoring and daytime sleepiness, while poor sleep quality is associated with insomnia. Therefore, PLWH who experience snoring or excessive sleepiness should be referred for further investigation of OSA during clinical examinations. Additionally, PLWH who experience insomnia should be examined for good sleep quality, and appropriate interventions should be considered if necessary. Furthermore, since increasing age is associated with a higher risk of sleep problems, healthcare providers should prioritize sleep problem assessments among older PLWH.

Moreover, as the prevalence of sleep disorders increases, mental health problems such as anxiety and depression also tend to increase in PLWH. Depression is commonly associated with poor sleep quality and insomnia, while anxiety is associated with poor sleep quality, insomnia, and sleep apnea. Therefore, mental health interventions may help alleviate insomnia and poor sleep quality in PLWH. Policymakers, healthcare providers, and other stakeholders should work towards improving mental health interventions to enhance sleep quality among PLWH.

## Data Availability

It’s possible for us to share out data. To access the data and materials, please contact the corresponding author, Dr. Akbarpour.

## References

[1] Sankri-Tarbichi AG (2012). Obstructive sleep apnea-hypopnea syndrome: Etiology and diagnosis. Avicenna J. Med..

[2] Ikeda M, Kaneita Y (2014). The newest epidemiology trend of insomnia. Nihon Rinsho..

[3] de la Vega R, Tomé-Pires C, Solé E, Racine M, Castarlenas E, Jensen MP (2015). The Pittsburgh sleep quality index: Validity and factor structure in young people. Psychol. Assess..

[4] Sateia MJ (2014). International classification of sleep disorders. Chest.

[5] George S, Bergin C, Clarke S, Courtney G, Codd MB (2016). Health-related quality of life and associated factors in people with HIV: An Irish cohort study. Health Quality Life Outcomes.

[6] Pujasari H, Levy J, Culbert G, Steffen A, Carley D, Kapella M (2021). Sleep disturbance, associated symptoms, and quality of life in adults living with HIV in Jakarta, Indonesia. AIDS Care.

[7] Rogers BG, Lee JS, Bainter SA, Bedoya CA, Pinkston M, Safren SA (2020). A multilevel examination of sleep, depression, and quality of life in people living with HIV/AIDS. J. Health Psychol..

[8] Sidibé, M. Programme on HIV/AIDS-understanding fast-track accelerating action to end the aids epidemic by 2030: UNAIDS; 2015 [Available from: https://www.unaids.org/sites/default/files/media_asset/201506_JC2743_Understanding_FastTrack_en.pdf.

[9] UNAIDS. Global HIV & AIDS statistics 2021 [cited 2021. Available from: https://www.unaids.org/en/resources/fact-sheet.

[10] Jabbari F, Dabaghzadeh F, Khalili H, Abbasian L (2015). Associated factors of sleep quality in HIV-positive individuals. Future Virol..

[11] Robbins JL, Phillips KD, Dudgeon WD, Hand GA (2004). Physiological and psychological correlates of sleep in HIV infection. Clin. Nurs. Res..

[12] Wu J, Wu H, Lu C, Guo L, Li P (2015). Self-reported sleep disturbances in HIV-infected people: A meta-analysis of prevalence and moderators. Sleep Med..

[13] Najafi A, Mahboobi M, Sadeghniiat Haghighi K, Aghajani F, Nakhostin-Ansari A, Soltani S (2021). Sleep disturbance, psychiatric issues, and employment status of Iranian people living with HIV. BMC Res. Notes.

[14] Asgari S, Najafi A, Sadeghniiat K, Gholamypour Z, Akbarpour S (2021). The association between body mass index and risk of obstructive sleep apnea among patients with HIV. J. Res. Med. Sci..

[15] Aswathappa J, Garg S, Kutty K, Shankar V (2013). Neck circumference as an anthropometric measure of obesity in diabetics. N. Am. J. Med. Sci..

[16] Buysse DJ, Reynolds CF, Monk TH, Berman SR, Kupfer DJ (1989). The Pittsburgh Sleep Quality Index: A new instrument for psychiatric practice and research. Psychiatry Res..

[17] Tan A, Yin JD, Tan LW, van Dam RM, Cheung YY, Lee C-H (2017). Using the Berlin questionnaire to predict obstructive sleep apnea in the general population. J. Clin. Sleep Med..

[18] Srijithesh PR, Shukla G, Srivastav A, Goyal V, Singh S, Behari M (2011). Validity of the Berlin Questionnaire in identifying obstructive sleep apnea syndrome when administered to the informants of stroke patients. J. Clin. Neurosci..

[19] Ghanei Gheshlagh R, Hemmati Moslek M, Baghi V (2014). A study on the relation between body mass index and sleep apnea in patients suffering diabetes type2. J. Diabetes Nurs..

[20] Sadeghniiat-Haghighi K, Yazdi Z, Kazemifar AM (2016). Sleep quality in long haul truck drivers: A study on Iranian national data. Chin. J. Traumatol..

[21] Sadeghniiat Haghighi K, Montazeri A, Khajeh Mehrizi A, Aminian O, Rahimi Golkhandan A, Saraei M (2013). The Epworth sleepiness scale: Translation and validation study of the Iranian version. Sleep Breath.

[22] Yazdi Z, Sadeghniiat-Haghighi K, Zohal MA, Elmizadeh K (2012). Validity and reliability of the Iranian version of the insomnia severity index. Malays. J. Med. Sci..

[23] Yazdi Z, Sadeghniiat-Haghighi K, Zohal MA, Elmizadeh K (2012). Validity and reliability of the Iranian version of the insomnia severity index. Malays. J. Med. Sci..

[24] Samani S, Jokar B (2008). Validity and reliability short-form version of the depression, anxiety and stress. J. Soc. Sci. Humanit. Univ. Shiraz.

[25] Sahebi A, Asghari MJ, Salari RS (2006). Validation of depression anxiety and stress scale (DASS-21). Dev. Pscychol..

[26] Johns MW (2000). Sensitivity and specificity of the multiple sleep latency test (MSLT), the maintenance of wakefulness test and the Epworth sleepiness scale: Failure of the MSLT as a gold standard. J. Sleep Res..

[27] Dabaghzadeh F, Khalili H, Ghaeli P, Alimadadi A (2013). Sleep quality and its correlates in HIV positive patients who are candidates for initiation of antiretroviral therapy. Iran. J. Psychiatry.

[28] Bisong E (2017). Predictors of sleep disorders among HIV out-patients in a tertiary hospital. Recent Adv. Biol. Med..

[29] Allavena C, Guimard T, Billaud E, de la Tullaye S, Reliquet V, Pineau S (2014). Prevalence and risk factors of sleep disturbances in a large HIV-infected adult population. J. Int. AIDS Soc..

[30] Bedaso A, Abraham Y, Temesgen A, Mekonnen N (2020). Quality of sleep and associated factors among people living with HIV/AIDS attending ART clinic at Hawassa University comprehensive specialized Hospital, Hawassa, SNNPR, Ethiopia. Plos one.

[31] Rodríguez Estrada E, Iglesias Chiesa MC, Fresán Orellana A, Reyes-Terán G (2018). Factors associated with poor sleep quality among HIV-positive individuals in Mexico City. Salud Mental.

[32] Legas G, Beyene GM, Asnakew S, Belete A, Desie T (2022). Poor sleep quality and associated factors among HIV-positive pregnant women in Northwest, Ethiopia: A facility-based, cross-sectional study. BMC Psychiatr..

[33] Junaid K, Ali H, Khan AA, Khan TA, Khan AM, Khan A (2021). Prevalence and associated factors of depression among patients with HIV/AIDS in Lahore, Pakistan: Cross-sectional study. Psychol. Res. Behav. Manag..

[34] Luu BR, Nance RM, Delaney JA, Ruderman SA, Heckbert SR, Budoff MJ (2022). Brief Report: Insomnia and risk of myocardial infarction among people with HIV. JAIDS J. Acquir. Immune Defic. Syndr..

[35] Cruess DG, Antoni MH, Gonzalez J, Fletcher MA, Klimas N, Duran R (2003). Sleep disturbance mediates the association between psychological distress and immune status among HIV-positive men and women on combination antiretroviral therapy. J. Psychosom. Res..

[36] Crum-Cianflone NF, Roediger MP, Moore DJ, Hale B, Weintrob A, Ganesan A (2012). Prevalence and factors associated with sleep disturbances among early-treated HIV-infected persons. Clin. Infect. Dis..

[37] White JL, Darko DF, Brown SJ, Miller JC, Hayduk R, Kelly T (1995). Early central nervous system response to HIV infection: Sleep distortion and cognitive-motor decrements. Aids.

[38] Wibbeler T, Reichelt D, Husstedt I-W, Evers S (2012). Sleepiness and sleep quality in patients with HIV infection. J. Psychosom. Res..

[39] Faraut B, Malmartel A, Ghosn J, Duracinsky M, Leger D, Grabar S (2018). Sleep disturbance and total sleep time in persons living with HIV: A cross-sectional study. AIDS Behav..

[40] Taibi DM (2013). Sleep disturbances in persons living with HIV. J. Assoc. Nurses AIDS Care.

[41] Njoh AA, Mbong EN, Mbi VO, Mengnjo MK, Nfor LN, Ngarka L (2017). Likelihood of obstructive sleep apnea in people living with HIV in Cameroon–preliminary findings. Sleep Sci. Pract..

[42] Patil SP, Schneider H, Schwartz AR, Smith PL (2007). Adult obstructive sleep apnea: Pathophysiology and diagnosis. Chest.

[43] McNicholas WT (2009). Obstructive sleep apnea and inflammation. Prog. Cardiovasc. Dis..

[44] Chaput J-P, Katzmarzyk PT, LeBlanc AG, Tremblay MS, Barreira TV, Broyles ST (2015). Associations between sleep patterns and lifestyle behaviors in children: An international comparison. Int. J. Obes. Suppl..

[45] Hyle EP, Martey EB, Bekker L-G, Xu A, Parker RA, Walensky RP (2021). Diet, physical activity, and obesity among ART-experienced people with HIV in South Africa. AIDS Care.

[46] Willig AL, Webel AR, Westfall AO, Levitan EB, Crane HM, Buford TW (2020). Physical activity trends and metabolic health outcomes in people living with HIV in the US, 2008–2015. Progr. Cardiovasc. Dis..

[47] Twigg HL, Knox KS (2013). Impact of antiretroviral therapy on lung immunology and inflammation. Clin. Chest Med..

[48] Chen Y-C, Lin C-Y, Li C-Y, Zhang Y, Ko W-C, Ko N-Y (2020). Obstructive sleep apnea among HIV-infected men in the highly active antiretroviral therapy era: A nation-wide longitudinal cohort study in Taiwan, 2000–2011. Sleep Med..

[49] Zicari S, Sessa L, Cotugno N, Ruggiero A, Morrocchi E, Concato C (2019). Immune activation, inflammation, and non-AIDS co-morbidities in HIV-infected patients under long-term ART. Viruses.

[50] Kim M, Um Y-H, Kim T-W, Kim S-M, Seo H-J, Jeong J-H (2021). Association between age and sleep quality: Findings from a community health survey. Sleep Med. Res..

